# High cardiovascular risk of patients with type 2 diabetes is only partially attributed to angiographic burden of atherosclerosis

**DOI:** 10.1177/1479164120953612

**Published:** 2020-09-22

**Authors:** Simone Battermann, Andrea Milzi, Rosalia Dettori, Kathrin Burgmaier, Nikolaus Marx, Mathias Burgmaier, Sebastian Reith

**Affiliations:** 1 Department of Cardiology, University Hospital of the RWTH Aachen, Aachen, Germany; 2 Department of Pediatrics, University Hospital of Cologne, Koln, Nordrhein-Westfalen, Germany

**Keywords:** Quantitative coronary angiography, coronary artery disease, Type 2 diabetes mellitus, Gensini score, COURAGE score

## Abstract

**Background:**

Patients with type 2 diabetes (T2DM) are at high risk for cardiovascular events and present more severe coronary artery disease (CAD). The Gensini and COURAGE scores are established angiographic instruments to assess CAD severity, which may also predict future cardiovascular risk. However, it is unclear if these scores are able to depict the increased risk of patients with T2DM and stable CAD (T2DM-SAP).

**Methods:**

We performed quantitative coronary angiography and assessed the Gensini and COURAGE scores in 124 patients with T2DM-SAP. Angiographic data were compared to patients with stable angina without T2DM (Non-DM-SAP, *n* = 74), and to patients with acute coronary syndrome and T2DM (T2DM-ACS, *n* = 53).

**Results:**

T2DM-SAP patients had similar Gensini and COURAGE-scores compared to Non-DM-SAP-patients (Gensini: 14.44 ± 27.34 vs 11.49 ± 26.99, *p* = 0.465; COURAGE: 3.48 ± 4.49 vs 3.60 ± 4.72, *p* = 0.854). In contrast, T2DM-SAP patients had significantly lower Gensini (14.44 ± 27.34 vs 30.94 ± 48.74, *p* = 0.003) and lower COURAGE (3.48 ± 4.49 vs 5.30 ± 4.63, *p* = 0.016) scores compared to T2DM-ACS-patients.

**Conclusion:**

Both the Gensini and the COURAGE score fail to predict the high cardiovascular risk of patients with T2DM-SAP. Therefore, these scores should be used with caution in the assessment of future risk of patients with T2DM. However, among T2DM-ACS patients, both scores are increased, reflecting the high cardiovascular risk in this patient population.

## Introduction

Patients with type 2 diabetes mellitus (T2DM) have an enhanced risk to develop coronary artery disease (CAD) with higher disease-related morbidity and mortality rates compared to patients without TD2M.^1–5^ Specifically, patients with T2DM without a previous history of myocardial infarction (MI) have a similar risk of subsequent cardiovascular events as patients without T2DM but with a history of MI.^2^ Moreover, a recent meta-analysis assessing 12 randomized trials demonstrated that patients with T2DM suffer significantly more often from recurrent cardiac events following percutaneous coronary intervention, including target lesion re-revascularization, re-hospitalization, MI and in-stent-restenosis.^6^

Several groups have established scores to quantify the extent of CAD as determined by quantitative coronary angiography (QCA). Among these, one of the most comprehensive is the Gensini score, which takes into consideration not only the luminal narrowing through a plaque but also the localization of the stenosis.^7^ More recently, in a sub-study of the COURAGE trial, Mancini et al. elaborated a further score system assessing the anatomic burden of coronary atherosclerosis as determined by QCA.^8^ This so called COURAGE score could consistently predict death, MI and non-ST-elevation acute coronary syndromes (ACS), differently from the extent of the ischemic myocardium as assessed by nuclear cardiology imaging.^8^

It is current knowledge derived from both pathology and intravascular imaging studies that patients with T2DM yield a more vulnerable plaque phenotype compared to patients without T2DM, including a thinner fibrous cap,^9–13^ a larger necrotic lipid core^9,14^ and a more extensive macrophage infiltration.^9,12^ However, the relationship between T2DM and the angiographic extent of macrovascular coronary atherosclerosis remains currently unclear. In an angiographic study assessing severity and extent of coronary atherosclerosis using the Gensini score, Pajunen et al. found no significant difference between patients with versus without T2DM.^15^ Similarly, Kataoka et al. revealed no relevant effect of the presence of T2DM regarding the Gensini Score.^16^ In contrast, Chen et al. identified T2DM as a relevant predictor of severe CAD, as defined by a Gensini Score > 20.^17^ Hence, there is an ongoing controversy whether this score adequately depicts the increased cardiovascular risk of CAD in patients with T2DM. To the best of our knowledge, until now no study yet employed the COURAGE Score to assess the impact of T2DM on coronary atherosclerosis.

Therefore, the current study aimed to investigate the extent and severity of CAD, assessed with two established score systems, namely the Gensini and the COURAGE Score, in patients with T2DM with stable angina pectoris (SAP) compared to both (1) patients with SAP and no T2DM and to (2) patients with ACS and T2DM.

## Methods

### Study population

In this analysis we included 124 patients with T2DM and stable CAD (T2DM-SAP) that underwent a coronary angiography at the Department of Cardiology, University Hospital of the RWTH Aachen, Germany. The indication for angiography was based on clinical symptoms, presence of myocardial ischemia in stress-testing and/or electrocardiographic abnormalities. T2DM was defined as either previously diagnosed diabetes, treated by diet, ongoing antidiabetic therapy and/or an HbA1C ⩾ 6.5%. Stable CAD was defined as no progression of symptoms within the last 6 weeks.

This population was compared to two control groups: (1) 74 patients with stable angina and without T2DM (Non-DM-SAP), and (2) 53 patients with ACS and T2DM (T2DM-ACS). ACS was defined as ST-elevation myocardial infarction (STEMI), non-ST-elevation myocardial infarction (NSTEMI) or unstable angina, characterized by angina at rest or progressive angina within the last 6 weeks without elevated biochemical markers. Exclusion criteria were left main disease, hemodynamic or rhythmologic instability, renal insufficiency (defined as a serum creatinine>1.5 mg/dl), heart failure with severe left ventricular disfunction (defined as ejection fraction<35%), pregnancy and age <18 years. Clinical history and baseline characteristics as well as laboratory testing were performed in all patients prior to coronary angiography.

All patients signed written informed consent to the study protocol and answered a detailed questionnaire concerning clinical characteristics. The study was approved by the local Ethics Committee and is in accordance with the Declaration of Helsinki on ethical principles for medical research investigating human subjects.

### Quantitative coronary angiography

Standardized QCA was performed using off-line imaging analysis on a validated QCA software (Philips Inturis Cardio View, QCA V3.3, Pie Medical imaging, Eindhoven, Netherlands). Angiographic analysis was performed on a frame showing the coronary segment and the stenosis in diastole, in full length, without shortening due to angle and, if possible, without overlap from other vessels. The coronary catheter (5F) or guiding catheter (6F) were used for calibration. Analysis of QCA data included reference lumen diameter (RD), minimal lumen diameter (MLD), percent diameter stenosis and stenosis length. MLD was defined as the smallest diameter in the chosen segment if there was a stenosis (>25% narrowing of the vessel lumen). RD was taken from the mean of proximal and distal reference diameter at the proximal and distal segment of the stenosis. The percent diameter stenosis was derived from the RD and the MLD and calculated as ([RD]-[MLD])/RD. Stenosis length was defined as the segment around the MLD with a percent diameter stenosis of at least 50% compared with the predefined RD.

### Gensini score

The Gensini score is designed to quantify the coronary stenosis severity of the entire coronary artery system by dividing the coronary tree into 10 segments and evaluating the luminal narrowing and the anatomic location of each coronary stenosis. Therefore, both the lumen diameter and the angiographic presentation of concentric or eccentric lesions were assessed. As described in the original paper by Gensini et al.,^7^ luminal narrowings of 25, 50, 75, 90 and 99% as well as total occlusion of a coronary vessel were allocated to a Gensini score of 1, 2, 4, 8, 16 and 32, respectively. These numbers were multiplied by weighting factors determined according to the anatomical localization of the stenosis. Segments with a diameter of less than 1,5mm were excluded from analysis. The final Gensini score was calculated as the sum of the adjusted results.

### COURAGE score

The COURAGE score is based on the quantification of the anatomic burden of coronary artery disease in the recently published COURAGE trial.^8^ Coronary angiograms were assessed for the presence of stenoses ⩾50% in the major epicardial vessels and primary branches. Patients with less than 50% diameter stenosis were allocated as negative for coronary vessel disease. By distinguishing between the proximal and non-proximal segment of the coronary artery, different combinations of single-, double-, and triple-vessel disease were described anatomically, thereby creating an anatomic burden score as a continuous variable from 0 to 17, as previously described.^8^ Per definition the COURAGE trial excluded patients with left main coronary artery disease.

### Statistical analysis

All statistical analyses were performed with SPSS Statistics (IBM Corp., Armonk, NY, USA). Categorical variables are expressed as n and percentage. Analysis of variance (ANOVA) with the LSD test for post-hoc analysis was used to compare continuous variables. Pearson’s χ^2^ test was used to compare nominal variables and p-values were not adjusted for multiple comparisons. A p-value < 0.05 was regarded as statistically significant.

## Results

### Baseline characteristics

The analysed group of 124 T2DM-SAP patients did not differ significantly from the two control groups with regard to age and gender distribution. Both groups of patients with T2DM had higher incidences of hypertension, dyslipidaemia, a higher body mass index, a longer history of smoking, a higher LDL-cholesterol as well as a lower HDL-cholesterol compared to non-T2DM patients. Clinical and baseline characteristics including comparative data between study groups are presented in Table 1.

**Table 1.** Baseline characteristics.

	T2DM SAP	Non-DM-SAP	T2DM-SAP versus Non-DM-SAP	T2DM-ACS	T2DM-SAP versus T2DM-ACS
	(*n* = 124)	(*n* = 74)	*p*-value	(*n* = 53)	*p*-value
Age (years)	67.8 ± 8.6	67.1 ± 11.1	0.624	69.6 ± 8.8	0.259
Male Gender (*n*, %)	83 (66.9)	45 (60.8)	0.383	35 (66.0)	0.908
BMI (kg/m2)	31.1 ± 5.2	27.3 ± 4.3	0.016	29.5 ± 5.9	0.056
Hypertension (*n*,%)	110 (88.7)	52 (72.2)	<0.001	43 (81.8)	0.177
MAP	97.5 ± 13.7	86.4 ± 21.7	<0.001	93.2 ± 11.6	0.109
Dyslipidemia (*n*, %)	90 (72.6)	37 (51.4)	0.003	33 (62.3)	0.172
Pack years	18.5 ± 22.0	19.8 ± 27.8	0.698	19.3 ± 20.0	0.826
Family history (*n*, %)	59 (47.6)	36 (50.7)	0.172	17 (32.7)	0.069
HbA1C (mg/dl)	7.1 ± 1.3	5.68 ± 0.36	<0.001	7.5 ± 1.6	0.826
ß- blocker (*n*, %)	101 (81.5)	45 (64.3)	0.008	38 (73.1)	0.213
ACE- Inhibitor (*n*, %)	89 (73.6)	40 (57.1)	0.081	35 (67.3)	0.403
Metformin (*n*, %)	88 (71.5)	0	NA	31 (58.5)	0.090
Insulin (*n*, %)	44 (35.4)	0	NA	21 (39.6)	0.531
Sulfonylurea (*n*, %)	28 (22.6)	0	NA	6 (11.3)	0.082
Incretin based therapy (*n*, %)	23 (18.5)	0	NA	9 (17.0)	0.804
Statin (n, %)	82 (66.1)	48 (64.9)	0.061	33 (62.3)	0.295
Total cholesterol (mg/dl)	192.2 ± 41.5	200.9 ± 51.1	0.186	189.4 ± 41.1	0.710
HDL- cholesterol (mg/dl)	43.5 ± 10.7	53.0 ± 23.8	<0.001	44.1 ± 13.8	0.827
LDL- cholesterol (mg/dl)	118.2±33.8	134.2± 42.3	0.004	115.3 ± 35.2	0.645
Triglycerides (mg/dl)	197.9 ± 120.9	141.6 ± 71.7	<0.001	163.6 ± 90.3	0.052
Serum Creatinine (mg/dl)	1.0 ± 0.3	1.0 ± 0.2	0.244	1.0 ± 0.3	0.441
Fasting glucose (mg/dl)	161.3 ± 51.9	102.1 ± 11.9	<0.001	165.0 ± 69.6	0.666
Known CAD (*n*, %)	46 (37.1)	21 (29.2)	0.018	16 (30.2)	0.378
Prior MI (*n*, %)	25 (20.2)	17 (23.9)	0.410	12 (22.6)	0.645
Prior PCI (*n*, %)	37 (29.8)	19 (26.4)	0.110	14 (26.4)	0.710

SAP: Stable angina pectoris; T2DM: Type 2 Diabetes mellitus; ACS: acute coronary syndrome; NS: not significant; BMI: Body Mass Index; NSTEMI: Non-ST-Elevation Myocardial Infarction; PCI: Percutaneous Coronary Intervention; CAD: Coronary Artery Disease; MI: Myocardial Infarction; ACE: angiotensin converting enzyme; ARB: angiotensin receptor blocker; ASA: acetyl- salicylate- acid; HDL: high density lipoprotein; LDL: low density lipoprotein. NA: not available; NS: non significant.

The data are presented as mean ± SD or *n* (%).

### Gensini- and Courage-score

T2DM-SAP patients showed a Gensini score of 14.44 ± 27.34 and a COURAGE score of 3.48 ± 4.49. Both scores were not significantly different when comparing these patients to the Non-DM-SAP control group (Gensini: 14.44 ± 27.34 vs 11.49 ± 26.99, *p* = 0.465; COURAGE: 3.48 ± 4.49 vs 3.60 ± 4.72, *p* = 0.854). In contrast, T2DM-SAP patients had a significantly lower Gensini (14.44 ± 27.34 vs 30.94 ± 48.74, *p* = 0.003) and lower COURAGE-score (3.48 ± 4.49 vs 5.30 ± 4.63, *p* = 0.016) compared to T2DM-ACS-patients.

We further subdivided both groups of patients with T2DM according to insulin use and to adequacy of glucose control (HbA1c ⩽ or >7.0%) Neither quality of glucose control nor type of anti-diabetic medication revealed a statistical difference regarding the severity of CAD as assessed by the Gensini and COURAGE scores. For further detail, see Tables 2 and 3.

**Table 2.** The Gensini- and Courage-Score in patients with type 2 diabetes – difference between patients with and without insulin therapy.

	T2DM-SAP *n* = 124			T2DM-ACS *n* = 53		
	No insulin therapy *n* = 80	Insulin therapy *n* = 44	P	No insulin therapy *n* = 32	Insulin therapy *n* = 21	*p*
Gensini score	12.81 ± 23.63	17.47 ± 33.26	0.370	35.58 ± 56.43	23.88 ± 33.98	0.398
Courage score	2.88 ± 4.10	4.63 ± 5.05	0.054	5.34 ± 5.15	5.23 ± 3.81	0.936

**Table 3.** The Gensini- and Courage-Score in patients with type 2 diabetes – difference between adequate (HbA1c<7.0%) and inadequate (HbA1c>7.0%) glucose control.

	T2DM-SAP *n* = 124			T2DM-ACS *n* = 53		
	HbA1C ⩽ 7% *n* = 74	HbA1C > 7% *n* = 50	*p*	HbA1C ⩽ 7% *n* = 25	HbA1C > 7% *n* = 28	*p*
Gensini Score	12.18 ± 20.44	17.74 ± 35.04	0.316	35.75 ± 63.22	26.31 ± 29.37	0.493
Courage Score	3.57 ± 4.76	3.36 ± 4.10	0.802	4.68 ± 4.75	5.74 ± 4.55	0.487

## Discussion

The main findings of the current investigation are:

1. T2DM-SAP patients do not show any significant difference in the severity of CAD as assessed by both the Gensini and the COURAGE scores when compared to Non-DM-SAP patients.
2. However, T2DM-SAP present a less extensive CAD than T2DM-ACS-patients.

T2DM is a confirmed risk factor of CAD and its sequelae, ACS and cardiovascular death. Many investigators have reported that patients with T2DM present a greater severity of CAD,^9–14^ resulting in high morbidity and mortality rates.^1–6^ However, the assessment of the impact of T2DM on the extent of CAD measured through QCA-based scores lead to conflicting results.^15–17^ Furthermore, it is currently unclear if scores, such as the Gensini and COURAGE score, are suitable to depict the high cardiovascular risk of patients with T2DM.

In the present study we extend the current knowledge by demonstrating no significant difference in the angiographic severity of atherosclerosis among patients with SAP independently of the presence of T2DM. These results are in line with smaller previous studies^15,16^ but yet surprising, given that angiographic scores may predict future cardiovascular events,^8^ which are known to be heavily correlated with T2DM.^1–5^ In the light of our data, it is tempting to speculate that the QCA-based scores describing the extent of CAD in patients with SAP may not be able to depict the well-known incremental cardiovascular risk caused from T2DM.^1–6^ It may be therefore hypothesized that this increased risk in the population with T2DM arises from morphologic aspects of CAD other than its mere anatomic burden. One of these aspects can be found in the more vulnerable plaque phenotype present in plaques of patients with SAP and T2DM, in particular with lower fibrous cap thickness,^9–13^ more extensive necrotic lipid core^9,14^ and higher rate of macrophage infiltration^9,12^ in comparison to patients without T2DM. Thus, our data suggest that angiographic scores have to be used with caution to assess future cardiovascular risk in SAP patients with T2DM. Furthermore, to the incremental risk of patients with T2DM may contribute also the presence of coronary microvascular disease,^18^ which is not detected by coronary angiography and therefore is not depicted from QCA-based scores as the ones considered.

In both groups of patients with T2DM and irrespective of clinical presentation (SAP or ACS), no significant differences could be found between the two considered scores in dependence of insulin therapy or quality of glycaemic control. Whereas an insufficient glycaemic control in previous studies showed only limited effects on cardiovascular events,^19^ these data are especially interesting considering that patients under insulin therapy represent a subpopulation with an even higher cardiovascular risk as compared to other patients with T2DM.^20^ The absence of significant differences in the scores in dependence of insulin therapy or quality of glycaemic control may highlight a diagnostic restriction of these scores, which do not allow the identification of these high-risk subgroups. On the contrary, other investigations have indicated that intravascular imaging may provide more detailed information on these patients, at least for the quality of glycaemic control, which according to a study from Kuroda et al. significantly affects features of plaque vulnerability as fibrous cap thickness, dimensions of the necrotic lipid core and macrophage infiltration.^21^ Furthermore, although the quality of glycaemic control and insulin therapy do not seem to be associated with higher scores reflecting a more extensive CAD, they are still able to influence the cardiovascular complications and, in general, the overall prognosis of patients with T2DM through a variety of effects which are not limited to atherosclerosis and plaque vulnerability, but also include a prothrombotic status^22^ and a more vulnerable myocardium.^23^

Recently, cardioprotective effects of antidiabetic drugs such as SGLT2-inhibitors^24,25^ or GLP-1 agonists^26^ were described. However, these effects cannot explain the findings of our study, that is, the similar angiographic scores between patients with and without T2DM, since these cardio-protective medication were not yet sufficiently diffused at the time of study inclusion. In fact, no patient was on SGLT2-inhibitors and only 18.1% of patients with T2DM (18.5% in patients with stable CAD, 17.0% in patients with ACS) presented an incretin-based therapy, which however was in most cased represented by the DPP-4 inhibitor sitagliptin, which did not show any cardiovascular benefit in the TECOS study.^27^ The effects of these potent cardioprotective medications on angiographic burden of atherosclerosis need to be evaluated in further analyses.

Furthermore, we could measure an angiographically more advanced CAD in T2DM-ACS patients compared to T2DM-SAP patients. This may be partly due to the so called *diabetic paradox* highlighted by Niccoli et al., that is, the presentation of ACS only in a more advanced stadium of CAD in patients with T2DM.^28^ However, given that the anatomic burden of CAD has been shown to be predictive for future cardiovascular events,^8^ our data may suggest an enhanced future cardiovascular risk for patients with diabetes following MI compared to patients with T2DM and SAP. These findings are in line with epidemiological studies, which previously demonstrated among patients with T2DM a striking increase in future cardiovascular events following a previous MI.^2^ However, the future risk of cardiovascular events is not barely limited to the atherosclerotic burden, as suggested by previous works using intravascular imaging indicating that plaques of T2DM-ACS patients present a more vulnerable phenotype compared to stable patients,^29–31^ which may play a complementary role to the higher atherosclerotic burden in the worse prognosis of this population.

### Limitations

Although we currently present the largest population in which the angiographic extent of CAD was assessed using both the Gensini and the COURAGE score, we cannot exclude a potential selection bias due to the study design. In spite of the comprehensive analysis of coronary macrovessel disease with QCA and QCA-based scores, we did not assess microvascular disease, which may be a relevant factor in the worse prognosis of patients with T2DM. Furthermore, this study compared T2DM-SAP patients to both Non-DM-SAP and T2DM-ACS control groups; patients without T2DM and with ACS were not included in the current study, which may limit its generalization. A possible confounding factor in our analysis may be the higher risk profile of patients with T2DM, such as a higher prevalence of dyslipidemia and hypertension as well as a higher BMI, compared to patients without T2DM; although this is known from previous literature, it may partly influence the extent of the atherosclerotic burden and therefore our results. Moreover, although the negative prognostic effects of T2DM and previous ACS are known through previous literature, in our study we cannot draw any direct conclusion on this point due to the absence of follow-up data. Of course, although we could detect a similar severity of CAD between stable patients with and without T2DM, it has to be noticed that the higher cardiovascular risk of T2DM patients is not exclusively a function of the extent of atherosclerosis, but arises rather from a multitude of concurring factors including a pro-thrombotic milieu^22^ and a vulnerable myocardium^23^ – factors that cannot be assessed due to the design of our study.

## Conclusion

Our data suggest that both T2DM and non-T2DM patients with SAP show similar severity of CAD as assessed by angiographic scores. On the other hand, patients with T2DM and ACS display a more advanced coronary atherosclerosis compared to T2DM-SAP patients. Our findings indicate that angiographic scores may reflect the higher risk of T2DM-ACS patients compared to T2DM-SAP patients, but also that they only incompletely assess the higher risk profile of T2DM-SAP patients in comparison to Non-DM-SAP. This incremental risk of patients with T2DM in SAP seems to arise mostly from a more vulnerable plaque phenotype rather than from a more extensive CAD. In order to assess this high-risk-phenotype, intravascular imaging is essential. We conclude that angiographic scores should be used with caution to assess future cardiovascular risk in patients with SAP and T2DM.

## References

[1] Kannel WB McGee DL. Diabetes and cardiovascular disease. The Framingham study. JAMA 1979; 241(19): 2035–2038. 430798 10.1001/jama.241.19.2035

[2] Haffner SM Lehto S Rönnemaa T, et al. Mortality from coronary heart disease in subjects with type 2 diabetes and in nondiabetic subjects with and without prior myocardial infarction. N Engl J Med 1998; 339: 229–234. 9673301 10.1056/NEJM199807233390404

[3] Malmberg K Yusuf S Gerstein HC, et al. Impact of diabetes on long-term prognosis in patients with unstable angina and non-Q-wave myocardial infarction: results of the OASIS (Organization to Assess Strategies for Ischemic Syndromes) Registry. Circulation 2000; 102(9): 1014–1019. 10961966 10.1161/01.cir.102.9.1014

[4] Shah AD Langenberg C Rapsomaniki E, et al. Type 2 diabetes and incidence of cardiovascular diseases: a cohort study in 1·9 million people. Lancet Diabetes Endocrinol 2015; 3(2): 105–113. 25466521 10.1016/S2213-8587(14)70219-0 PMC4303913

[5] Sarwar N Gao P Seshasai SR, et al. Diabetes mellitus, fasting blood glucose concentration, and risk of vascular disease: a collaborative meta-analysis of 102 prospective studies. Lancet 2010; 375(9733): 2215–2222. 20609967 10.1016/S0140-6736(10)60484-9 PMC2904878

[6] Li N Yang YG Chen MH. Comparing the adverse clinical outcomes in patients with non-insulin treated type 2 diabetes mellitus and patients without type 2 diabetes mellitus following percutaneous coronary intervention: a systematic review and meta-analysis. BMC Cardiovasc Disord 2016; 16(1): 238. 27887590 10.1186/s12872-016-0422-0 PMC5124234

[7] Gensini GG. A more meaningful scoring system for determining the severity of coronary heart disease. Am J Cardiol 1983; 51(3): 606. 6823874 10.1016/s0002-9149(83)80105-2

[8] Mancini GB Hartigan PM Bates ER, et al. Prognostic importance of coronary anatomy and left ventricular ejection fraction despite optimal therapy: assessment of residual risk in the Clinical Outcomes Utilizing Revascularization and Aggressive DruG Evaluation Trial. Am Heart J 2013; 166(3):481–487. 24016497 10.1016/j.ahj.2013.07.007

[9] Moreno PR Murcia AM Palacios IF, et al. Coronary composition and macrophage infiltration in atherectomy specimens from patients with diabetes mellitus. Circulation 2000; 102(18): 2180–2184. 11056089 10.1161/01.cir.102.18.2180

[10] Burke AP Kolodgie FD Zieske A, et al. Morphologic findings of coronary atherosclerotic plaques in diabetics: a postmortem study. Arterioscler Thromb Vasc Biol 2004; 24(7): 1266–1271. 15142859 10.1161/01.ATV.0000131783.74034.97

[11] Fukunaga M Fujii K Nakata T, et al. Multiple complex coronary atherosclerosis in diabetic patients with acute myocardial infarction: a three-vessel optical coherence tomography study. EuroIntervention 2012; 8(8): 955–961. 23253548 10.4244/EIJV8I8A145

[12] Kato K Yonetsu T Kim SJ, et al. Comparison of nonculprit coronary plaque characteristics between patients with and without diabetes: a 3-vessel optical coherence tomography study. JACC Cardiovasc Interv 2012; 5(11): 1150–1158. 23174639 10.1016/j.jcin.2012.06.019

[13] Milzi A Burgmaier M Burgmaier K, et al. Type 2 diabetes mellitus is associated with a lower fibrous cap thickness but has no impact on calcification morphology: an intracoronary optical coherence tomography study. Cardiovasc Diabetol 2017; 16(1): 152. 29195505 10.1186/s12933-017-0635-2 PMC5709861

[14] Marso SP Mercado N Maehara A, et al. Plaque composition and clinical outcomes in acute coronary syndrome patients with metabolic syndrome or diabetes. JACC Cardiovasc Imaging 2012; 5(3 Suppl): S42–S52. 22421230 10.1016/j.jcmg.2012.01.008

[15] Pajunen P Nieminen MS Taskinen MR, et al. Quantitative comparison of angiographic characteristics of coronary artery disease in patients with noninsulin-dependent diabetes mellitus compared with matched nondiabetic control subjects. Am J Cardiol 1997; 80(5): 550–556. 9294980 10.1016/s0002-9149(97)00420-7

[16] Kataoka Y Puri R Hammadah M, et al. Spotty calcification and plaque vulnerability in vivo: frequency-domain optical coherence tomography analysis. Cardiovasc Diagn Ther 2014; 4:460–469. 25610803 10.3978/j.issn.2223-3652.2014.11.06 PMC4278040

[17] Chen ZW Chen YH Qian J-Y, et al. Validation of a novel clinical prediction score for severe coronary artery diseases before elective coronary angiography, PLoS One 2014; 9(4): e94493. 24714416 10.1371/journal.pone.0094493 PMC3979855

[18] von Scholten BJ Hasbak P Christensen TE, et al. Cardiac (82)Rb PET/CT for fast and non-invasive assessment of microvascular function and structure in asymptomatic patients with type 2 diabetes. Diabetologia 2016; 59: 371–378. 26526662 10.1007/s00125-015-3799-x

[19] Zheng J Cheng J Zhang Q, et al. Association between glycosylated hemoglobin level and cardiovascular outcomes in diabetic patients after percutaneous coronary intervention. Medicine (Baltimore). 2016; 95(19): e3696. 27175711 10.1097/MD.0000000000003696 PMC4902553

[20] Bundhun PK Li N Chen MH. Adverse cardiovascular outcomes between insulin-treated and non-insulin treated diabetic patients after percutaneous coronary intervention: a systematic review and meta-analysis. Cardiovasc Diabetol 2015; 14: 135. 26446829 10.1186/s12933-015-0300-6 PMC4597459

[21] Kuroda M Shinke T Sakaguchi K, et al. Association between daily glucose fluctuation and coronary plaque properties in patients receiving adequate lipid-lowering therapy assessed by continuous glucose monitoring and optical coherence tomography. Cardiovasc Diabetol 2015; 14: 78. 26062762 10.1186/s12933-015-0236-x PMC4480895

[22] Hess K. The vulnerable blood. Coagulation and clot structure in diabetes mellitus. Hamostaseologie 2015; 35(1): 25–33. 10.5482/HAMO-14-09-0039 25418205

[23] Bugger H Bode C. The vulnerable myocardium. Diabetic cardiomyopathy. Hamostaseologie 2015; 35(1): 17–24. 10.5482/HAMO-14-09-0038 25408270

[24] Zinman B Wanner C Lachin JM, et al. Empagliflozin, cardiovascular outcomes, and mortality in type 2 diabetes. N Engl J Med 2015; 373(22): 2117–2128. 26378978 10.1056/NEJMoa1504720

[25] Neal B Perkovic V Mahaffey KW, et al. Canagliflozin and cardiovascular and renal events in type 2 diabetes. N Engl J Med 2017; 377: 644–657. 28605608 10.1056/NEJMoa1611925

[26] Marso MP Daniels GH Brown-Frandsen K, et al. Liraglutide and cardiovascular outcomes in type 2 diabetes. N Engl J Med 2016; 375: 311–322. 27295427 10.1056/NEJMoa1603827 PMC4985288

[27] Green JB Bethel MA Armstrong PW, et al. Effect of sitagliptin on cardiovascular outcomes in type 2 diabetes. N Engl J Med 2015; 373(3): 232–242. 26052984 10.1056/NEJMoa1501352

[28] Niccoli G Giubilato S Di Vito L, et al. Severity of coronary atherosclerosis in patients with a first acute coronary event: a diabetes paradox. Eur Heart J 2013; 34(10): 729–741. 23186807 10.1093/eurheartj/ehs393

[29] Reith S Battermann S Hoffmann R, et al. Optical coherence tomography derived differences of plaque characteristics in coronary culprit lesions between type 2 diabetic patients with and without acute coronary syndrome. Catheter Cardiovasc Interv 2014; 84(5): 700–707. 24155115 10.1002/ccd.25267

[30] Reith S Milzi A Lemma ED, et al. Intrinsic calcification angle: a novel feature of the vulnerable coronary plaque in patients with type 2 diabetes: an optical coherence tomography study. Cardiovasc Diabetol 2019; 18(1): 122. 31551093 10.1186/s12933-019-0926-x PMC6760065

[31] Kato K Yonetsu T Kim SJ, et al. Nonculprit plaques in patients with acute coronary syndromes have more vulnerable features compared with those with non-acute coronary syndromes: a 3-vessel optical coherence tomography study. Circ Cardiovasc Imaging 2012; 5(4): 433–440. 22679059 10.1161/CIRCIMAGING.112.973701

